# A nature-based solution to a landfill-leachate contamination of a confined aquifer

**DOI:** 10.1038/s41598-021-94041-7

**Published:** 2021-07-21

**Authors:** Daniel Abiriga, Andrew Jenkins, Live S. Vestgarden, Harald Klempe

**Affiliations:** Department of Natural Sciences and Environmental Health, Faculty of Technology, Natural Sciences and Maritime Sciences, University of South-Eastern Norway, Gullbringvegen 36, 3800 Bø, Norway

**Keywords:** Environmental chemistry, Environmental impact, Environmental sciences, Hydrology

## Abstract

Remediation of groundwater from landfill contamination presents a serious challenge due to the complex mixture of contaminants discharged from landfills. Here, we show the significance of a nature-based solution to a landfill-contaminated aquifer in southeast Norway. Groundwater physicochemical parameters monitored for twenty-eight years were used as a proxy to infer natural remediation. Results show that concentrations of the major chemical variables decreased with time and distance until they tailed off. An exception to this was sulphate, which showed an increase, but apparently, exhibits a stationary phase. The water types were found to be most similar between samples from active landfill and post-closure stages, while samples from the stabilised stage showed a different water type. All the chemical parameters of samples from the stabilised stage were found to be within the Norwegian drinking water standards, except iron and manganese, which were only marginally above the limits, an indication of a possible recovery of this aquifer. The findings highlight the significance of natural attenuation processes in remediating contaminated aquifers and have significant consequences for future contamination management, where natural remediation can be viewed as an alternative worth exploring. This is promising in the wake of calls for sustainable remediation management strategies.

## Introduction

Contamination of groundwater due to human activities remains a global challenge. Of particular concern is leachate from landfills, which can pollute both surface water and groundwater^1,2^. This may negatively affect an ecosystem functioning, as leachates may contain both legacy and emerging contaminants^2–5^. Several factors influence the composition and concentration of contaminants discharged by landfills^6–8^ and the eventual degree of groundwater contamination, which in turn shapes the resident microbial composition responsible for degradation^9^. These factors include landfill age, moisture content of the waste, amount of rainfall received, the nature of the underlying geological material, and the waste composition^6–8,10^.

Landfills operated in the seventies and earlier, before waste segregation was adopted, were fill-up with a mix of nearly anything^11^. Leachate composition from such landfills is highly variable and complex in nature, consisting of a mixture of contaminants^3,11–14^. This makes remediation of groundwater polluted by landfills even more costly and demanding than the remediation of hydrocarbon-polluted groundwater^11^. Therefore, passive remedial options, which utilise naturally occurring degradation, dilution and retardation processes (natural attenuation), are preferred over expensive conventional active remedial options such as the pump and treat techniques^15–18^. The impetus for natural attenuation, besides cost, lies in other merits of the technique such as its being efficient and nonintrusive to the environment, its wide applicability, and the absence of secondary wastes that would otherwise require an additional disposal stage^15–17,19^. Thus, natural attenuation has gained popularity in remediation of groundwater pollution from landfill leachate^12,20,21^. Newer remediation technologies are being developed, but are still in their infancy, with the majority being at laboratory scale^22^ with few field investigations^23^.

The main drawback of monitored natural attenuation is the time required to achieve remedial goals for the polluted environment^16,20^. The time frame is set by the regulatory body for pollution control and varies from country to country, with 30 years being common for non-hazardous wastes^15,24^. Such long-term remediation perspectives have significant financial implications. The highest expense is related to water quality monitoring, which may represent over 90% of the total cost^24^. Despite the cost, long-term monitoring helps to ensure that the attenuation capacity of the aquifer is not exceeded by the contaminant loads^14^. Providing unequivocal evidence of recovery of a contaminated aquifer requires that the contaminant loads be monitored in time and space for a period determined by the attainment of the minimum concentration of contaminants. This is evidenced by tailing off of the concentration of the monitored contaminants, which should approximate that quantified before the contamination occurred and/or that from a reference well located upstream of the contamination source. By this time, the landfill leachate may be considered to pose a minimal health risk^21^. Despite the tailing off, a potential risk may ensue if the contaminants tail off at levels above that of the reference value. In such scenarios tailing off cannot be equated to recovery. Also, the presence of chemical containers buried in landfills may undermine the perceived low risk^11^, due to a delayed leaching of contaminants as a result of corrosion of containers.

The high resource requirement associated with the implementation of long-term monitoring schemes remains a major limiting factor. This has hampered many monitored natural attenuation projects and led to few publications on successful natural remediation operations. In the present study, we report a study from a long-term monitoring of an aquifer in southeast Norway which was contaminated by a municipal landfill. The monitoring programme was followed for 28 years, starting from 1992, during active leaching of contaminants, to 2019 when the landfill was considered stabilised. The study aimed to (1) evaluate the long-term patterns in the groundwater quality, and (2) examine the changes in the groundwater chemistry as a function of the landfill stage and distance from the pollution source. Studies that have spanned so large a part of the lifetime of a landfill, and that have assessed the effect of different landfill stages on the receiving groundwater are scarce.

## Materials and methods

### Study area

The area is a glaciofluvial deposit at an elevation of 150 m above sea level at coordinates 59°25′58.26″ N and 9°06′1.53″ E. The mean air temperature of the study area varies from 15 °C in summer to − 2 °C in winter, with moderate temperatures of 6 °C and 7 °C occurring in spring and autumn respectively (https://seklima.met.no). The annual precipitation received in the area is 500–800 mm. Due to the nature of the area such as the occurrence of kettle holes and the distance from the urban centre, it was viewed as ideal for a landfill site for the municipality, and between 1974 and 1987, four landfill cells covering a total area of 30,137 square meters^25^ were opened and filled through 1974–1996. As an old landfill operated before waste segregation came into force, it received all kinds of waste typically generated from households, but wastewater treatment sludge, construction and demolition waste, and industrial waste were also deposited. The solid wastes came from Bø and Sauherad Municipalities (now merged to form Mid-Telemark Municipality). Neither liners for containment of leachate nor a leachate collection system were in place. The landfill was closed in February 1997 and the covered with 40–50 cm compacted clay^26^. The leachate from the landfill contaminated the underlying aquifer, and as required by regulation, the groundwater quality was monitored from the time of detection of pollution to the landfill post-closure. The aquifer is a subglacial river deposit with a matrix consisting of sand and gravel. It is confined by till (both as aquifer top and aquifer bottom) which is overlaid by a hard-packed moraine complex^27^. Both the bottom of the aquifer (bedrock) over which the till was deposited, and the aquifer walls are composed of Precambrian crystalline rock^26,28^. The mean transmittivity and the hydraulic conductivity from a pumping test at well R4 (see below) were 3.9 × 10^–3^ m^2^/s and 7 × 10^–4^ m/s, respectively, and the calculated groundwater flow velocity was 0.88 m/d^25^. The estimated mean aquifer recharge is 92 m^3^/d^28^. The aquifer was a source of drinking water to surrounding farms but due to the contamination, the use of the groundwater from the aquifer was discontinued.

**Figure 1:**
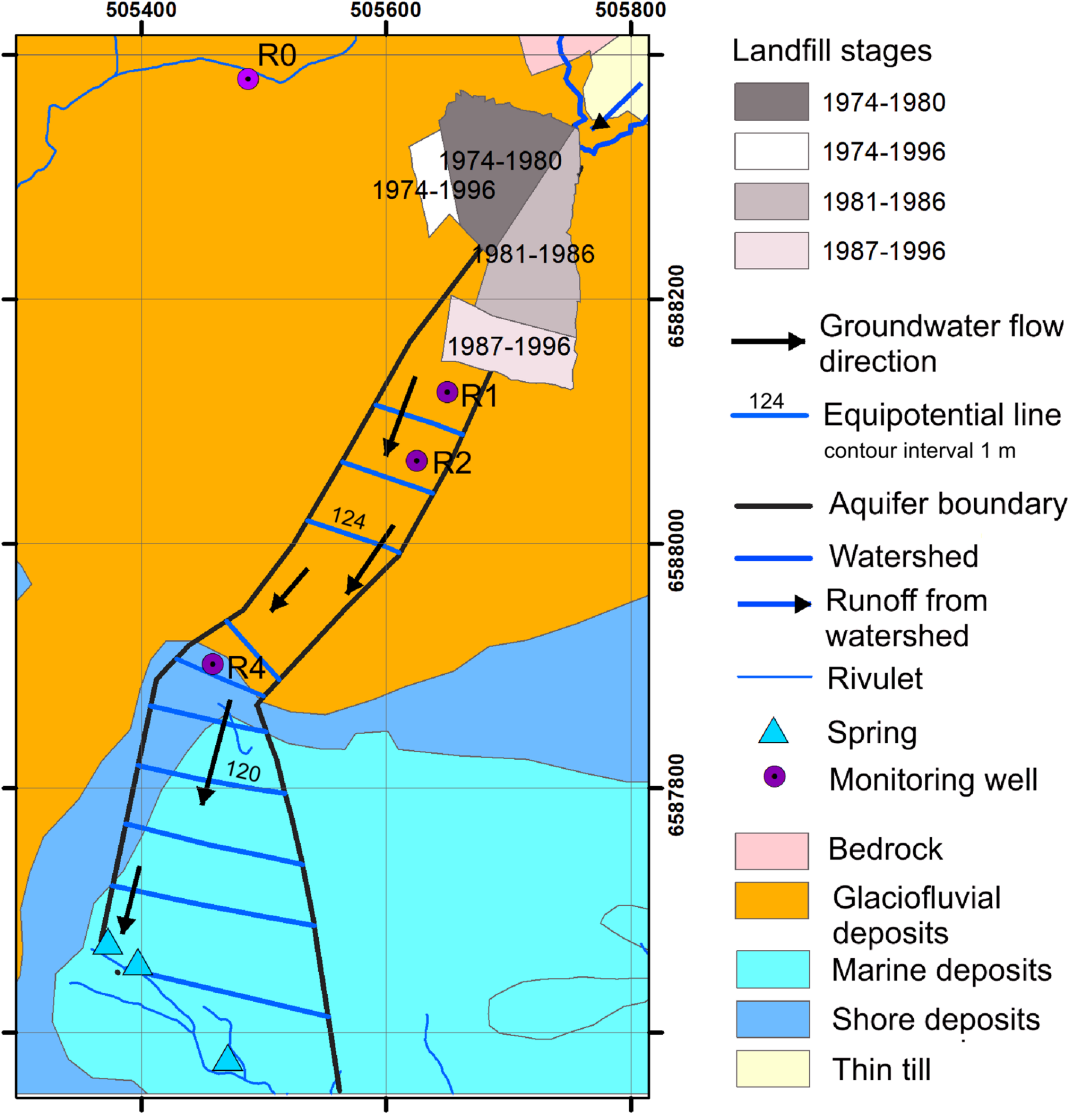
The study area showing the landfill, the site hydrogeology, and the location of the monitoring wells R0, R1, R2 and R4.

A monitoring scheme was developed to monitor the contamination and included three multilevel wells: R1 (five levels, at 126, 125, 124, 123 and 122 m.a.s.l), R2 (four levels, at 122, 121, 119 and 118 m.a.s.l) and R4 (three levels, at 118, 117 and 114 m.a.s.l) located in the contaminated aquifer downstream of the landfill (Fig. 1). The wells are respectively located at 26, 88 and 324 m from the edge of the landfill. In addition, well R0, which is located in the same geological formation but in a different (phreatic) aquifer, was used as a background well. The groundwater level in this well is ~ 3 m below the ground surface. Additional information on the site description including the hydrogeology is accessible elsewhere^25–28^.

### Experimental procedures

#### Groundwater sampling and chemical analysis

Groundwater samples were taken from the monitoring wells for the period 1992–2019. In the period 1992–2002, groundwater samples were collected quarterly, but during the period 2003–2019, samples were collected biannually. In addition, samples were collected from all the levels whenever possible in 1992–2006, but due to the associated cost of monitoring, the monitoring programme was revised to cover only two levels in each of the wells, except in 2018–2019 (again full-scale sampling was performed). The groundwater samples from R0 included in this study were collected in 2018 and 2019. Sampling was conducted in accordance with the ISO 5667–11 guideline, as described previously^25^.

Laboratory analyses were conducted following Norwegian Standards (NS) and/or ISO guidelines. The analyses were conducted for pH (NS 4720), conductivity (NS-ISO 7888), dissolve oxygen (NS 5813), nitrate (NS 4745), sulphate (ISO 10,304), ammonium (NS 4746), chloride (ISO 10,304), bicarbonate (NS-EN ISO 9963–2), total organic carbon (TOC) (NS 1484), calcium (NS-EN ISO 7980), potassium (NS-EN ISO 14,911), magnesium (NS-EN ISO 7980) and manganese (NS 4773). Iron, sodium, zinc and copper were determined using inductively coupled plasma atomic emission spectrometry (ICP-AES), while lead, chromium and cadmium were detected using inductively coupled plasma mass spectrometry (ICP-MS). Mercury was determined using cold vapour atomic fluorescence spectrometry (CV-AFS).

### Data analysis

Statistical analyses were conducted in R version 4.0.2^29^. The groundwater hydrochemical compositions among the sampling wells and the different landfill status were analysed using package hydrogeo^30^ and visualised using the R code obtained from github (https://gist.github.com/johnDorian/5561272). The groups (landfill status and well clusters) were tested for significant difference using the nonparametric Kruskal–Wallis test. The datasets used in this analysis were for the periods 1992–2003 and 2018–2019, because the major ions (calcium, magnesium, potassium, and bicarbonate), which form part of the input parameters to the hydrochemical model, were measured only during these periods. Two-dimensional contaminant profiles were constructed using package akima^31^ and were performed using a linear interpolation method. In this analysis, only chloride, sulphate, nitrate and bicarbonate for 2001 and 2019 were considered. These chemical variables were selected as they tend to be more mobile than their cationic counterparts^32^, while the years 2001 and 2019 were chosen because they had samples from all the levels in R1-R4. The groundwater parameters that were measured for the entire duration of monitoring were analysed for trends using Mann–Kendall trend test from package Kendall^33^, performed on the annual mean values.

## Results

### Changes in groundwater chemistry with time and distance

**Figure 2:**
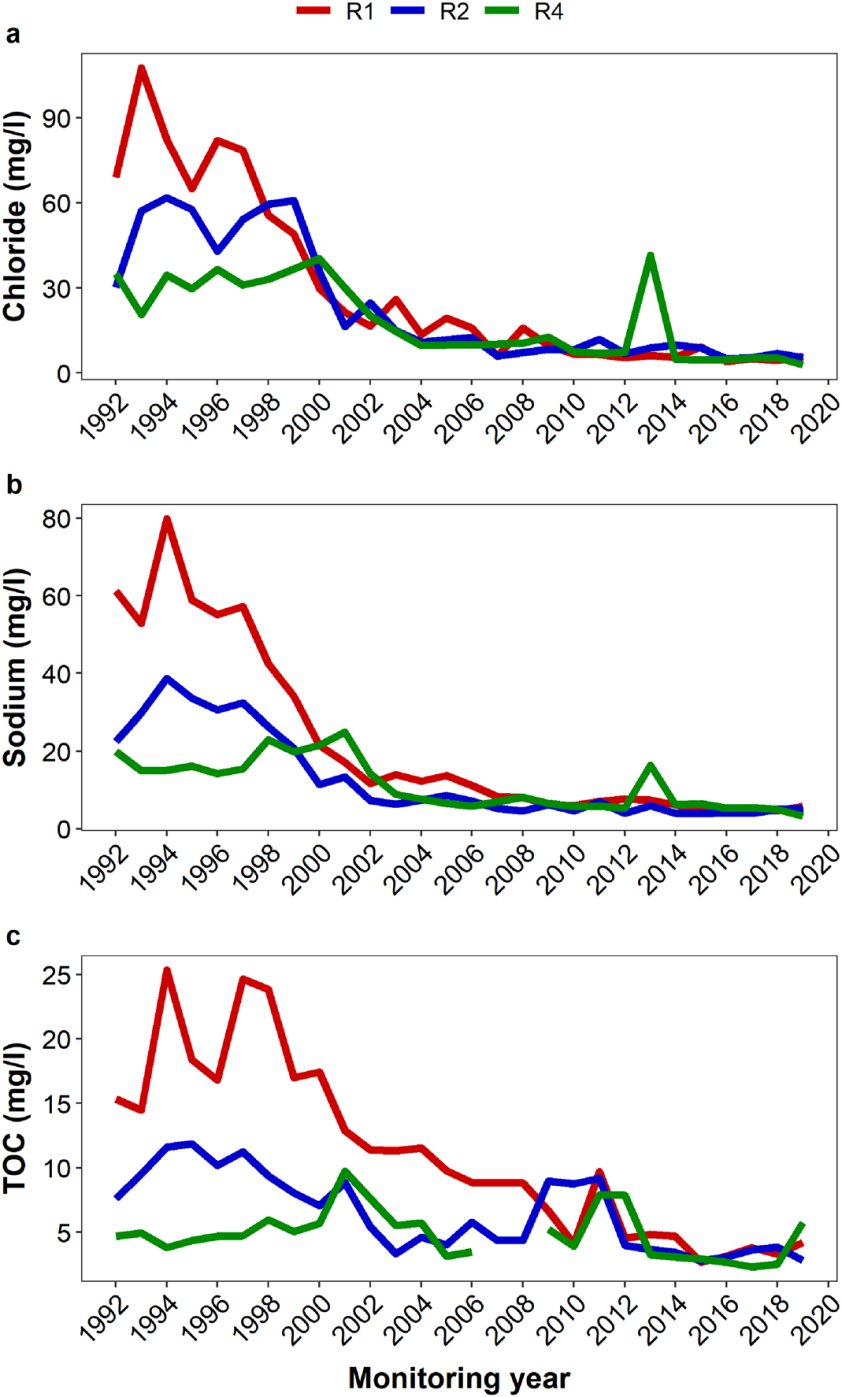
Long-term changes in annual mean values of chloride (**a**), sodium (**b**) and TOC (**c**) across the sampling wells R1, R2 and R4 from 1992 to 2019. The wells have been placed along the groundwater flow path in an increasing distance from the edge of the landfill.

**Figure 3:**
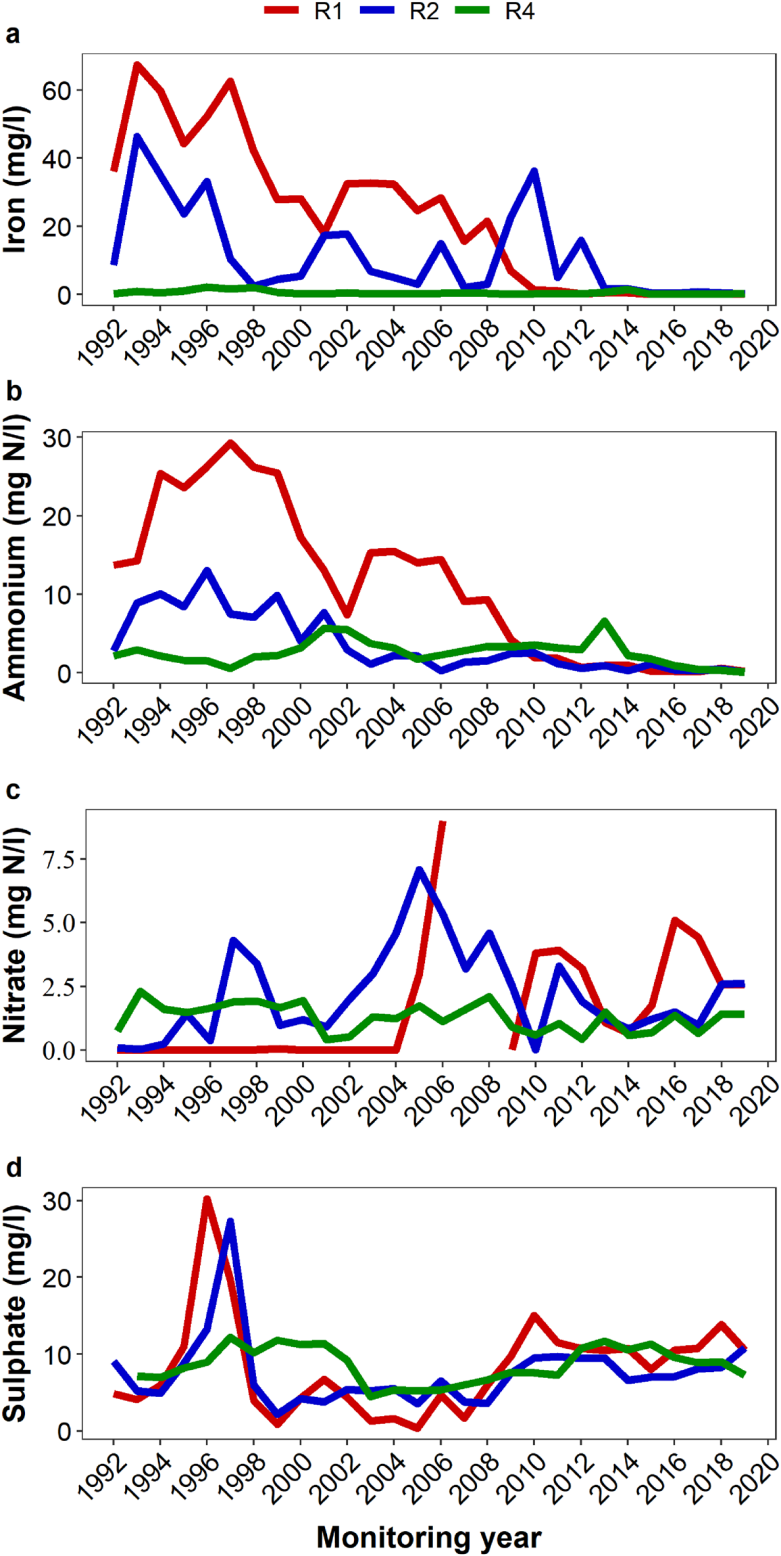
Long-term changes in annual mean values of iron (**a**), ammonium (**b**), nitrate (**c**) and sulphate (**d**) across the sampling wells R1, R2 and R4 from 1992 to 2019. The wells have been placed along the groundwater flow path in an increasing distance from the edge of the landfill.

The concentrations of all the major chemical variables decreased over time. Sodium and chloride tailed off as early as by 2010 (Fig. 2a-b). On the other hand, TOC (Fig. 2c), iron and ammonium (Fig. 3a-b) tailed off much later; by 2013 (iron), 2015 (TOC) and 2017 (ammonium). The oxidised chemical species and in particular, sulphate, showed a decrease immediately after the landfill closure in 1997, but only in R1 and R2 (Fig. 3d). From 2008, however, sulphate concentration increased gradually across the wells until it reached a plateau in 2012, when its concentration varied somewhat narrowly. Nitrate showed some complexity in its long-term leaching pattern, although recent records indicate an increase in its levels particularly in R1 (Fig. 3c). The concentrations of most of the solutes decreased along the flow path R1-R2-R4 (Supplementary Fig. S1 online). Sulphate and nitrate concentrations, however, increased along the groundwater flow direction. All the parameters showed a statistically significant difference across the wells (Supplementary Fig. S1 online).

Trend analysis as in Table 1 shows that both sulphate and nitrate are increasing in R1 and R2, while only sulphate shows an increasing trend in R4. However, the increase was significant only for nitrate and in R1. The rest of the parameters indicate decreasing trends in all the three wells. The decrease was strongest in R1, moderate in R2, and weak to moderate in R4.

**Table 1:** Mann–Kendall trend test results for parameters measured from 1992 to 2019.

	R1		R2		R4	
	τ	*p*-value	τ	*p*-value	τ	*p*-value
Sulphate	0.24	0.08	0.21	0.12	0.04	0.79
Chloride	− 0.82	** < 0.001**	− 0.69	** < 0.001**	− 0.62	** < 0.001**
Sodium	− 0.86	** < 0.001**	− 0.72	** < 0.001**	− 0.62	** < 0.001**
Iron	− 0.81	** < 0.001**	− 0.54	** < 0.001**	− 0.33	**0.02**
Nitrate	0.51	** < 0.001**	0.15	0.28	− 0.24	0.09
Ammonium	− 0.72	** < 0.001**	− 0.69	** < 0.001**	− 0.12	0.39
TOC	− 0.77	** < 0.001**	− 0.60	** < 0.001**	− 0.21	0.15

Significant results (*p* < 0.05) are indicated in bold face. R1, R2 and R4 are the monitoring wells located along the groundwater flow path in an increasing distance from the edge of the landfill. τ is the Kendall’s test statistic.

**Figure 4:**
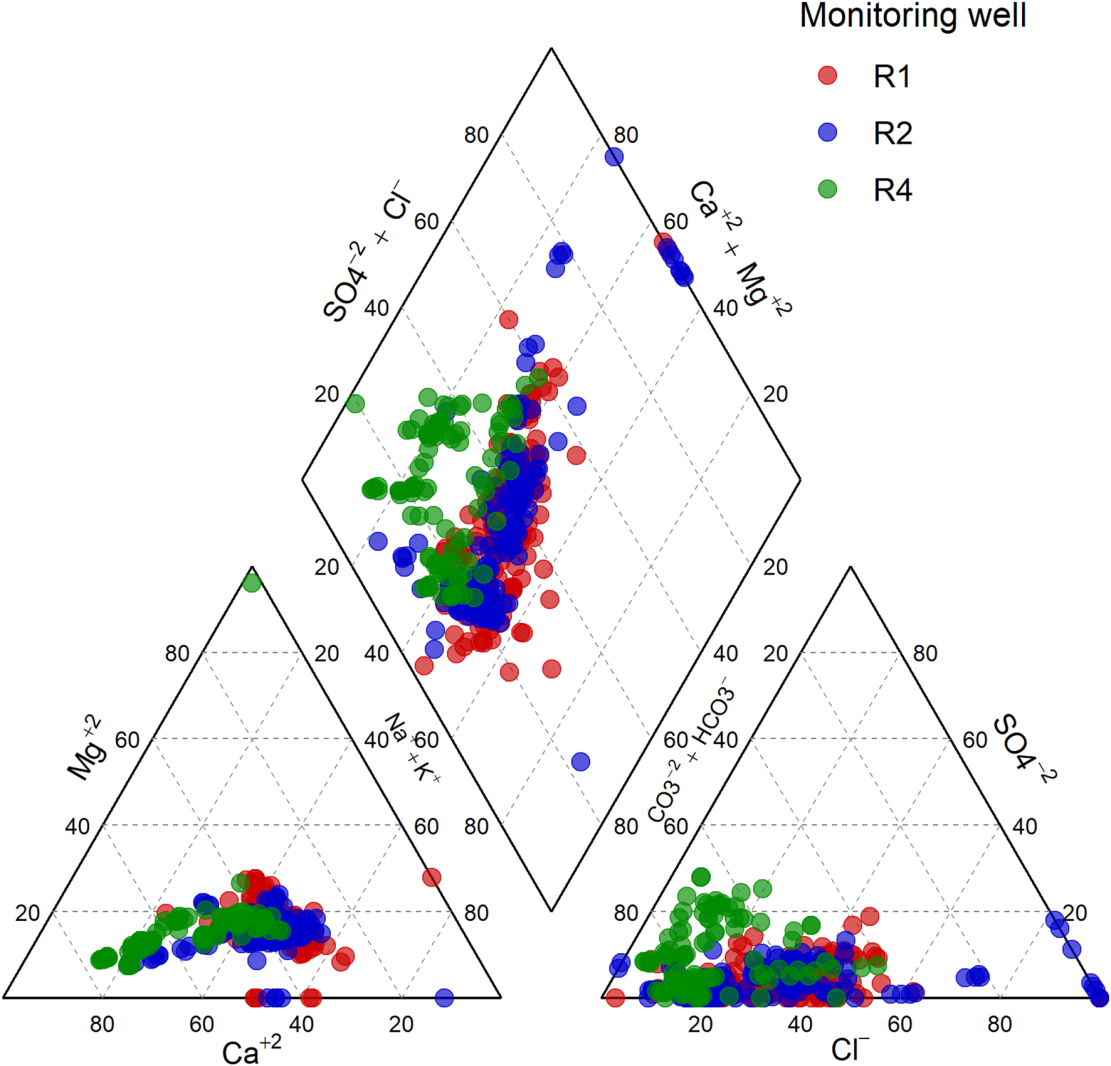
Characteristics of groundwater chemistry based on levels (% meq/l) of calcium, magnesium, potassium, sodium, chloride, sulphate and bicarbonate from the monitoring wells R1, R2 and R4. The wells were placed along the groundwater flow direction in an increasing distance from the edge of the landfill.

Based on the levels of the major ions (calcium, magnesium, potassium, sodium, chloride, sulphate and bicarbonate), the groundwater chemical composition was most similar between R1 and R2 (Fig. 4). Water from these wells was characterised by higher sodium + potassium and calcium, whereas R4 was richer in calcium. While the groundwater across the monitoring wells was enriched in bicarbonate, chloride was on few occasions quantified in higher levels in R2. R1 and R2 were predominantly characterised by three water types: Ca-(HCO_3_)_2_ type, Ca-Na-HCO_3_ type and Ca-Na-Cl type, in decreasing order. Occasionally, however, Na-HCO_3_, Na-Cl and Ca-Cl_2_ type waters were also measured. In R4, by contrast, Ca-(HCO_3_)_2_ type water predominated, while a few samples exhibited Ca-Na-Cl type water.

### Changes in groundwater chemistry relative to the landfill status

**Figure 5:**
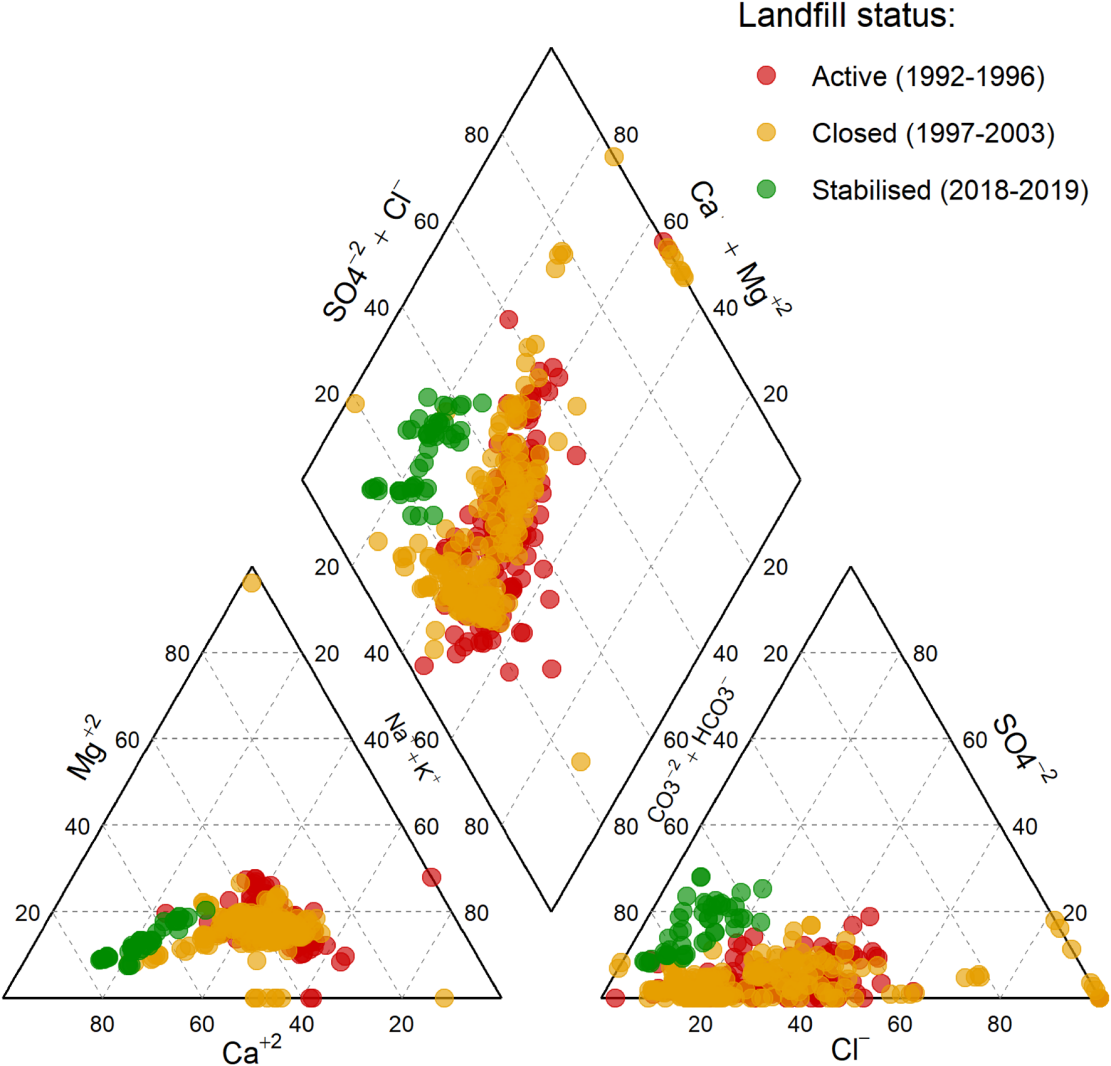
Characteristics of groundwater chemistry based on levels (% meq/l) of calcium, magnesium, potassium, sodium, chloride, sulphate and bicarbonate categorised by the landfill status (active, closed and stabilised). The years in parenthesis denote the periods for which groundwater chemistry data for classifying water types were available; otherwise, “closed” should span the period 1997–2016, while “stabilised” should cover 2017–2019.

Changes in groundwater chemistry as a function of the landfill status (Fig. 5) showed that the water chemistry was most similar between active and closed landfill phases. The samples were mostly characterised by high sodium + potassium and bicarbonate, but occasionally higher levels of chloride were observed. These samples composed three predominant water types: Ca-(HCO_3_)_2_ type, Ca-Na-HCO_3_ type and Ca-Na-Cl type. However, Na-HCO_3_, Na-Cl and Ca-Cl_2_ type waters were occasionally encountered. By contrast, groundwater samples taken in 2018–2019 clustered separately from the pre- and post-closure samples. These samples were characterised by higher levels of calcium and bicarbonate and were all Ca-(HCO_3_)_2_ type water.

**Figure 6:**
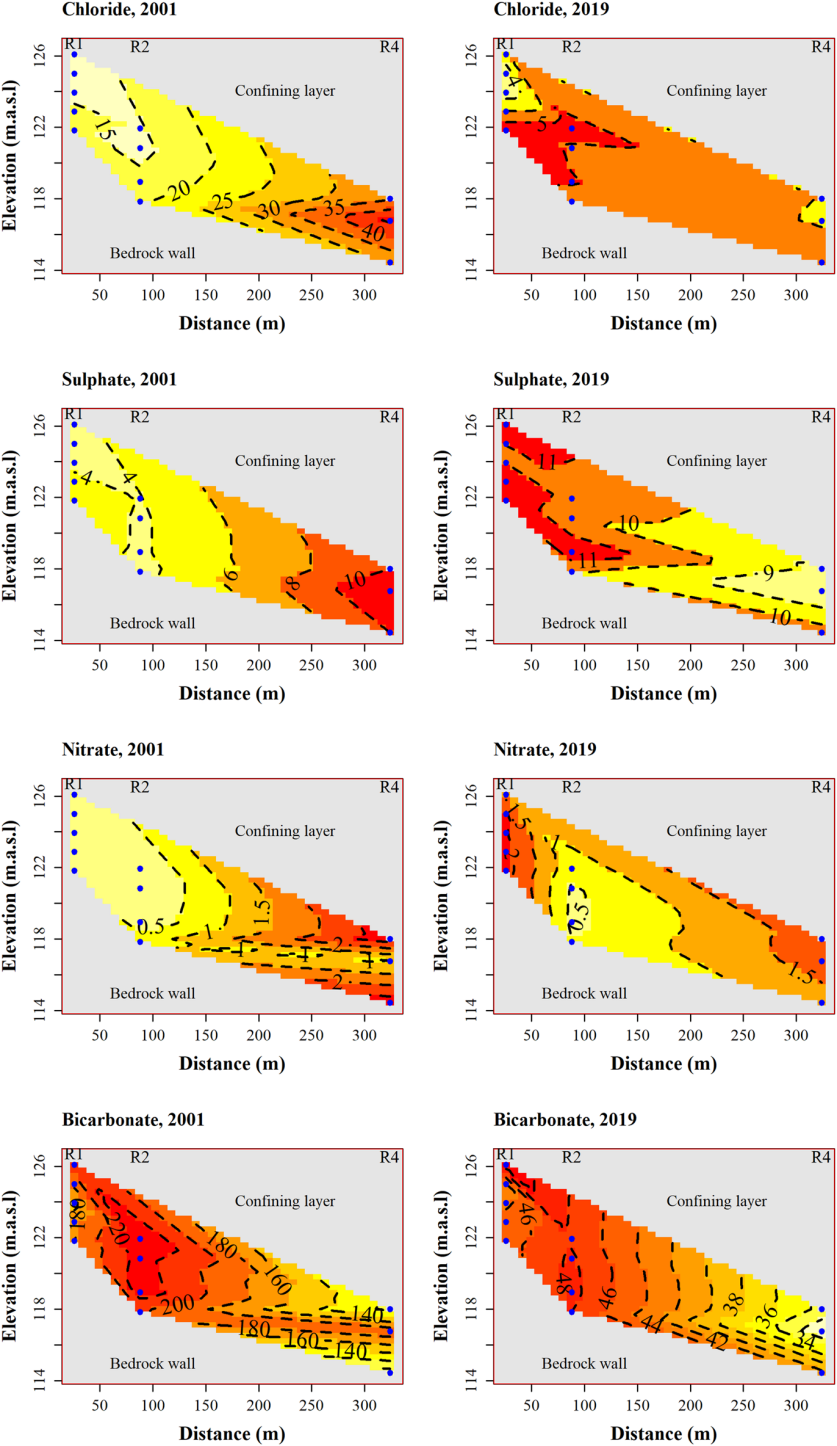
Depth-profiles of a few selected ions at two timepoints, November 2001 and October 2019, for chloride, sulphate, nitrate and bicarbonate. The blue dots depict the multilevel samplers in R1, R2 and R4. The wells have been placed along the groundwater flow path in an increasing distance from the edge of the landfill.

Depth-resolved profiles in Fig. 6 show changes in levels of chloride, sulphate, nitrate and bicarbonate in the aquifer in November 2001 and October 2019. In November 2001, a plume of chloride was moving out of the monitoring area, as evidenced by the higher measurements recorded in R4. By October 2019, however, chloride demonstrated a weaker gradient along the groundwater flow line and has decreased remarkably, with the highest measurement being ~ 5X lower than that measured in 2001. Both sulphate and nitrate registered higher concentrations in R4 in 2001, but in 2019, higher levels of these anions were detected in R1. The maximum concentration of both anions was similar between the two sampling campaigns, except that the patterns of distribution were reversed during the recent sampling campaign. Bicarbonate exhibited a similar pattern of distribution in 2001 and 2019. However, the actual concentrations differ greatly between the two timepoints, with the highest measurement recorded in the most recent sampling being lower by a factor of 4.6 (240/52).

The groundwater samples collected in 2018 and 2019, which represent samples under the stabilised landfill phase, were compared to the Norwegian drinking water standards and the background water quality from a nearby uncontaminated aquifer (R0). All the parameters except iron in R1, manganese in R1-R4, were below the limit for Norwegian drinking water standards (Table 2). Compared to the local groundwater quality, only pH, conductivity, dissolved oxygen, manganese and TOC in the contaminated aquifer occurred in levels greater or equal to that in R0.

**Table 2:** Comparison between the current groundwater quality under the stabilised landfill phase versus the Norwegian drinking water standards and the local background groundwater quality.

	R1	R2	R4	R0	Norwegian drinking water standards
pH	6.9	6.8	6.2	4.9	6.5–9.5
Conductivity (µS/cm)	223	136	160	35	2500
Dissolved oxygen	1.2	0.96	2.0	4.33	
Sulphate	12.2	9.5	8.1	3.0	250
Chloride	5.1	6.1	4.1	2.1	250
Nitrate (as N)	2.6	2.6	1.4	1.6	50
Bicarbonate	67	61	62	2.9	
Ammonium (as N)	0.37	0.29	0.2	ND	0.5
Sodium	5.2	5.1	4.1	1.8	200
Potassium	6.7	6.6	6.6	0.44	
Calcium	25	24	23	2.2	
Magnesium	2.9	2.9	3.0	0.51	
Manganese	0.21	0.24	0.5	0.51	0.05
Iron	0.03	0.27	0.15	ND	0.2
Zinc (µg/l)	NQ	NQ	NQ	NQ	
Copper (µg/l)	NQ	NQ	NQ	NQ	2000
Cadmium (µg/l)	0.09	0.03	0.07	NQ	5
Chromium (µg/l)	0.08	0.17	0.14	NQ	50
Lead (µg/l)	0.12	0.07	0.83	NQ	10
Mercury (ng/l)	0	0	0	NQ	1000
TOC	3.7	3.3	4.1	4.1	

Values are the means for samples collected in 2018 and 2019. All units are in mg/l, otherwise indicated. All values for mercury were below the limit of detection but reported as zero. Values for nitrate and ammonium in the Norwegian drinking water standards are in mg/l.

*ND* not detected (below the limit of detection); *NQ* not quantified.

## Discussion

Levels of the measured parameters varied differently throughout the monitoring period. Sodium and chloride declined and tailed off relatively earlier than the other ions. They also showed the strongest downward trends of − 0.86 and − 0.82, respectively (Table 1). Given that sodium is only slightly retarded while chloride is both nonreactive and almost not retarded at all^7,32^, the early tailing off of these two ions is suggestive of the depletion of leachable salts of sodium and chloride from the waste mass with age. The age of the landfill was found to be the most influential factor in explaining the levels of chloride measured in the groundwater^25^.

TOC and the reactive inorganic species such as iron and ammonium showed a delayed tailing off. While iron might be leached directly from the waste mass, it could also originate from the dissolution of minerals in the underlying strata under the influence of leachate. The source of the pollutant would be identified easily if there was data on the raw leachate, which could provide information on the source loading. The TOC and ammonium, on the other hand, are believed to originate from the landfill as degradative products of the resident microorganisms. Regardless of the source and type, these solutes are more reactive than the monovalent ions (sodium and chloride)^32^ and depending on the prevailing redox conditions, their mobility can be strongly retarded^26,34^. Therefore, this may cause delayed release of these solutes into the groundwater. It is also possible that recalcitrant nitrogenous and organic compounds might sustain the prolonged leaching of these pollutants, although such sources may only be minor, given the overall low concentrations of the contaminants. Sulphate and nitrate are the other reactive chemical species and unlike the other solutes, they were found to have upward trends in R1-R4 (sulphate) and R1-R2 (nitrate). This may be attributed to oxidation of metal sulphide and reduced nitrogen species in the landfill, due to a transition from reducing to oxidising condition^26^ as a result of the landfill stabilisation^8,35^. The microbiome of the aquifer has been found to harbour a wide range of biogeochemical cyclers, including iron oxidisers, sulphide oxidisers and ammonia oxidisers etcetera^36,37^. These microbes may be viewed as being involved in various oxidation and reduction processes. It is believed that the decrease in concentration of inorganic ions is primarily due to leaching of salts from the waste body, while the decline in levels of organic matter is due to attenuation mechanisms such as biodegradation, volatilisation and sorption^38,39^.

R1 (the proximal well), which is 26 m from the edge of the landfill, was the most polluted. In moving from the proximal well through R2 (the intermediate well) to R4 (the distal well), the concentrations of most of the ions decreased. An exception to this pattern was observed with nitrate and sulphate. The decrease in levels of the solutes with distance along the groundwater flow direction may be ascribed to dilution, sorption, complexation, precipitation and biodegradation^25,26^. The pattern observed with nitrate and sulphate could be attributed to oxidation of reduced compounds of the two elements along the groundwater flow path as dissolved oxygen increased along the groundwater flow path (Table 2 and Supplementary Fig. S6 online).

The piper diagram (Fig. 4) indicates that the water chemistry composition in both the proximal and intermediate wells were variable, consisting of six different water types. This suggests that the groundwater was under constant influence from the landfill leachate, causing the hydrochemistry to be transient. The episodic occurrence of chloride-rich water type observed in these wells might be attributed to the uneven leaching pattern which characterises landfill leachates^34^. Samples from the proximal and intermediate wells co-clustered, suggesting that they have similar water chemistry. However, the parameters indicated significant differences across the wells (Supplementary Fig. 1S online). The water type in the distal well was predominantly Ca-(HCO_3_)_2_. A previous study using piezometric analysis has reported a similar finding^21^. Natural attenuation in the aquifer is so substantial that the contaminant loads at the distal well have always been low. As evidenced from the trend analysis, the strength of downward trends decreased from the proximal to the distal wells, implying that the decay of contaminants has been minimal at the distal well. It is therefore not unlikely that the hydrochemistry of the distal well is dominated by a single hydrochemical facies. Because the composition of leachates may be complex^3,11–14^, the inferred water type may not reflect the true water–rock interaction and unambiguous interpretation is therefore, difficult.

Samples collected when the landfill was active (1992–1996) and after closure (1997–2003) clustered together, suggesting they have similar water chemistry, although the individual parameters showed significant difference across the three stages of the landfill (Supplementary Fig. S4 online). Up to six different water types, both single and mixed types characterised the active/closure stages (Fig. 5). Based on chloride concentration, it was previously observed that there was a change in the leaching pattern from random prior to landfill closure to less random after the landfill closure, and that closing the landfill significantly affected the groundwater quality^25^. However, no clear pattern between active and closed could be discerned from the major ions collectively (Fig. 5), suggesting that closing the landfill did not alter the relative compositions but rather altered the contaminant loads. The stabilised stage was, however, clearly distinct and composed only a single water type, Ca-(HCO_3_)_2_, illustrating that the leachable salts in the waste had been markedly depleted. Clearly, the hydrochemical facies recorded on landfill status mirrored that of distance (wells), which indicates recovery of the groundwater quality either as a function of landfill status or distance along flow path.

The most recent measurements indicate that nitrate and sulphate have increased in concentration across the wells. This reflects a transition to oxidising condition since 2009, and the fact that sulphate has reached a plateau is an indication of a stable oxidising environment in the landfill. This suggests that the landfill has attained its stabilised phase, during which more oxygen enters into the landfill than is depleted by microorganisms^8^. The oxygen would support additional oxidation of metal compounds within the landfill^35^, leading to the mobility of oxidised chemical species such as nitrate and sulphate. Both nitrate and sulphate have become mobile and less retarded in the aquifer, as evidenced by a weak gradient between the proximal and distal wells (Fig. 6). Because the landfill now leaches little reduced chemical species, there are insufficient electron donors to cause reduction of nitrate and sulphate to the extent comparable to the early years of monitoring. For example, iron and ammonium have since 2013 and 2017, respectively, become so low that the difference across the wells is virtually indiscernible (Fig. 3). Similarly, TOC has decreased remarkably at about the same time (2015) and fluctuated around 3 mg/l. Although detectable, the TOC may be inadequate to cause substantial reduction of nitrate and sulphate, or the TOC could be predominantly of recalcitrant compounds, as is expected of aged landfills^40^.

Heavy metals were unfortunately not monitored as consistently as the major chemical variables. Except for a few instances, the concentrations of most of the heavy metals were low (Supplementary Fig. S5 online). Previous studies have reported low concentrations of heavy metals in landfill leachates^1,25,38,41^ and it is now widely viewed that heavy metals do not constitute a serious pollution problem in municipal landfills^7,8,38,41,42^. On a long-term basis, heavy metals are thought to be precipitated in landfills under reducing condition, but at old age, oxygen is expected to intrude the landfill and cause an ecosystem shift from reducing to oxidising condition^8,35^. The transition leads to oxidation of previously precipitated heavy metals and results in a delayed release of trace elements into the environment^35^. So far, no excessive heavy metals were measured during the stabilised phase, but it remains to be seen if the anticipated delayed release will eventuate.

While there was lack of a clear trend in moving from the proximal through intermediate to the distal well, the maximum measured concentrations of chromium and copper were recorded from the distal well. A probable explanation could be the low pH in the distal well. The pH of the proximal and intermediate wells fluctuated within a narrow range at near-neutral (Supplementary Fig. S2 online), while measurements from the distal well remained relatively stable at ~ 5 over the monitoring period. pH is known to be one of the key influencers of metal speciation, with circum-neutral to high pH promoting metal precipitation and sorption, and lower pH triggering mobilisation^43^.

The goal of any remediation effort is full recovery of a contaminated environment. However, no data exists for the investigated aquifer before the contamination occurred and the tailing off could, therefore, not be unequivocally equated to full recovery. Instead, groundwater quality values were compared to the most stringent national quality standards, the Norwegian drinking water standards, and secondarily to a nearby well which should represent the local groundwater quality. Only manganese in the proximal–distal wells and iron in the intermediate well exceeded the Norwegian drinking water norms. Despite breaching the norms, these values were only marginally above the limits (under 1 mg/l) but have recovered from 99 and 16 mg/l for iron and manganese, respectively^26^. Clearly the groundwater is in the process of recovery, although it is arguable if the manganese will decrease lower than the current values attained in the respective wells, since a comparable concentration was recorded from the background well. Although within the drinking water limits, the highest pH value at present is 2 units above the local background value (Table 2), suggesting discharge of an alkaline leachate from the landfill. Likewise, the electrical conductivity, which is an indicator of the quantity of soluble salts in the groundwater, was within the guideline and decreased from > 1000 to < 250 µS/cm over the years (Supplementary Fig. S3 online), but apparently, the most recent measurement from the proximal well is about sixfold higher than that in the background well. This implies that there is still some active leaching from the landfill taking place. Since there will always be input coming from the buried waste, it is likely that neither the pH nor the conductivity will attain the natural background level. Landfills present continuous source loading^32^ that may take decades to centuries before substantial decay in concentration can be achieved^44^.

## Conclusion

Long-term groundwater chemical data for a period of 28 years was used to analyse for changes in water quality as a function of time, distance, and the stage of landfill stabilisation. Distance in this case was used to ascertain the significance of natural attenuation, which was found to be substantial. The results also showed that contaminant loads declined with time and reached a stationary minimum value, indicating depletion of leachable salts and suggests the attainment of a stabilised phase. The depletion of leachable salts is supported by the stabilised stage showing only a single hydrochemical facies as opposed to prior to stabilisation where six hydrochemical facies were found, suggesting discharge of a mixture of pollutants prior to the landfill stabilisation. Different pollutants attained the stabilised phase over different durations e.g., sodium and chloride after 19 years, iron 22 years, TOC 24 years and ammonium 26 years. The attainment of a stabilised phase with a concomitant substantial reduction in pollutant loading leading to tailing off of contaminants is indicative of the aquifer recovery. This highlights the applicability of non-invasive nature-based solutions to landfill-polluted aquifers.

## Supplementary Information

Supplementary Information.
