# Supplementary material for: A nature-based solution to a landfill-leachate contamination of a confined aquifer
Source: Sci Rep. 2021 Jul 21;11:14896. doi: 10.1038/s41598-021-94041-7 (PMC8295393; doi:10.1038/s41598-021-94041-7)
Supplement: Supplementary file 1 — Supplementary Information. [file 41598_2021_94041_MOESM1_ESM.pdf]

A nature-based solution to a landfill-leachate contamination of a confined aquifer

Daniel Abiriga*, Andrew Jenkins, Live S. Vestgarden and Harald Klempe

Department of Natural Sciences and Environmental Health, Faculty of Technology, Natural Sciences and Maritime Sciences, University of South-Eastern Norway, Bø, Norway.

* Corresponding author.

Department of Natural Sciences and Environmental Health, University of South-Eastern Norway, Gullbringvegen 36, NO-3800 Bø, Norway. Tel: +4735575378; Email: daniel.abiriga@usn.no

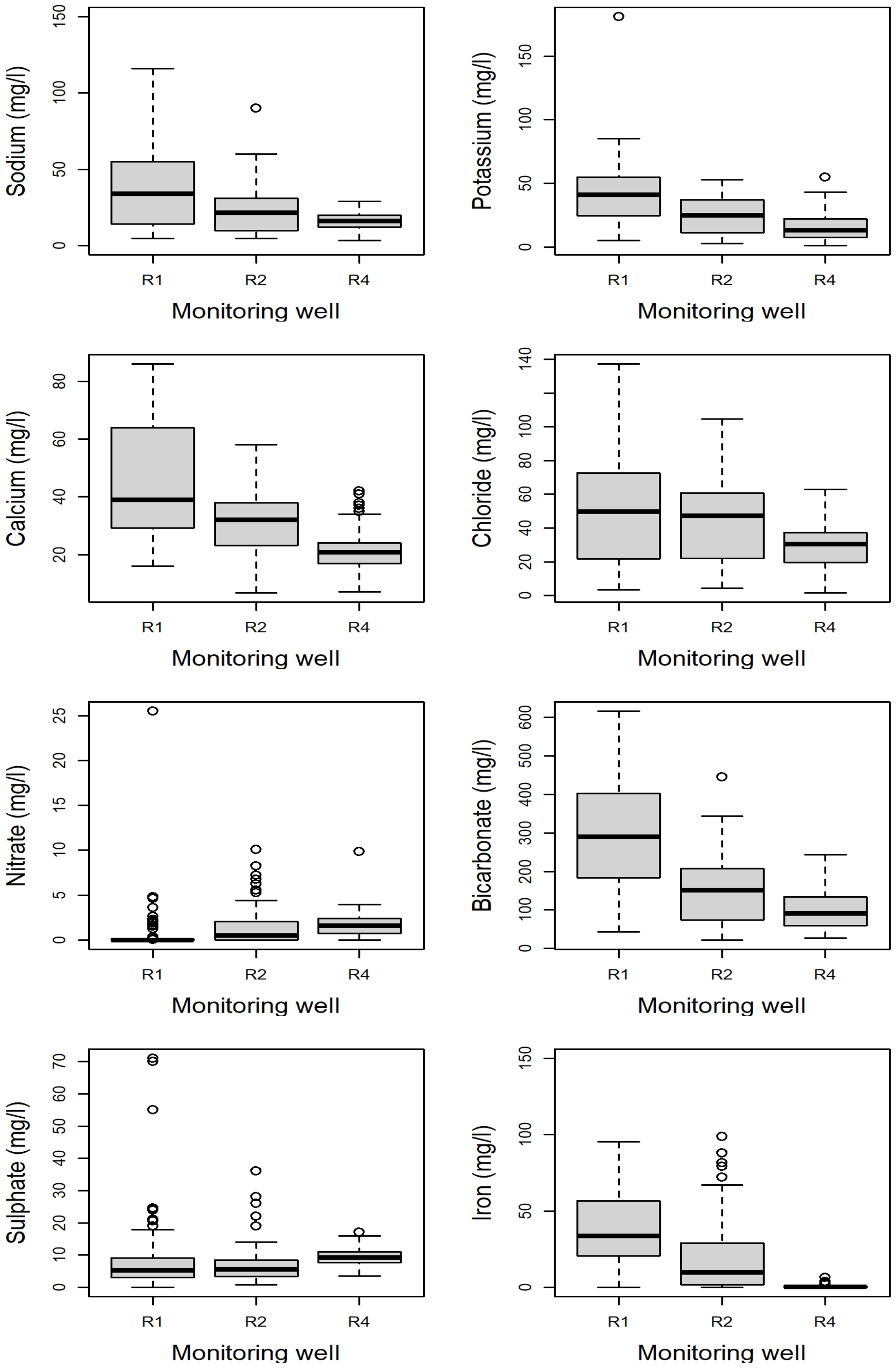

Figure S1. Changes in the groundwater quality across the sampling wells. The wells have been placed along the groundwater flow direction in an increasing distance from the edge of the landfill. All the parameters indicate significant differences. Sodium χ^2 = 54.3, df = 2, p = 1.652e-12; potassium χ^2 = 95.3, df = 2, p < 2.2e-16; calcium χ^2 = 137, df = 2, p < 2.2e-16; chloride χ^2 = 36.7, df = 2, p = 1.045e-08; nitrate χ^2 = 110, df = 2, p < 2.2e-16; bicarbonate χ^2 = 122, df = 2, p < 2.2e-16; sulphate χ^2 = 52.8, df = 2, p = 3.451e-12; iron χ^2 = 144, df = 2, p < 2.2e-16.

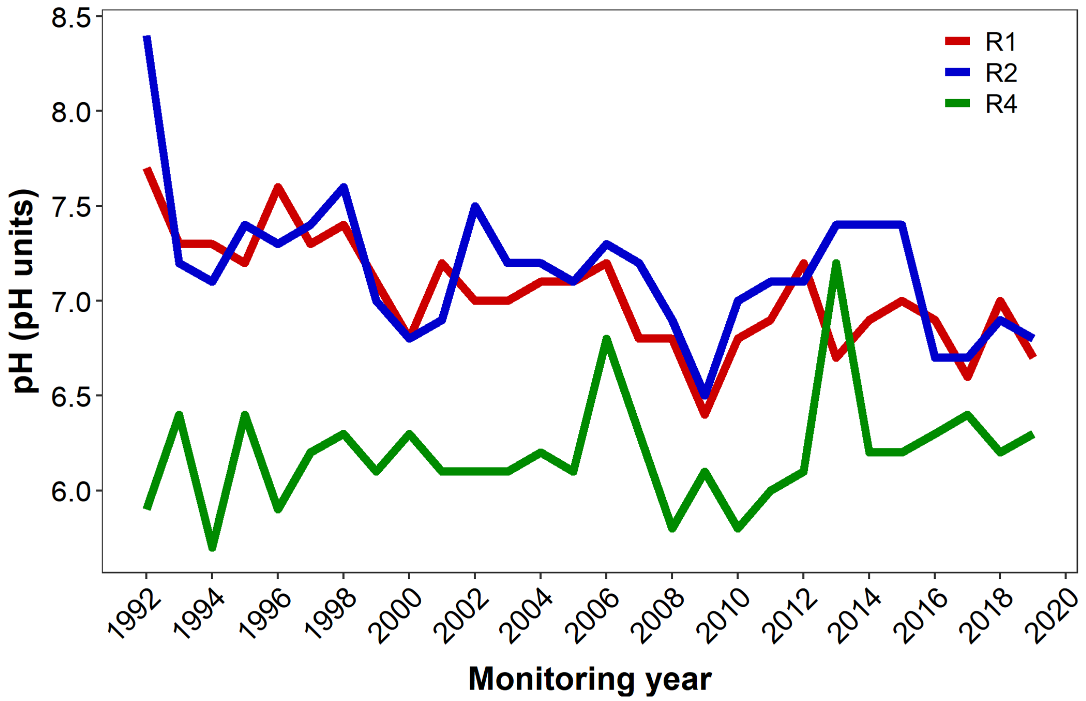

Figure S2. Long-term changes in annual mean values of pH across the sampling wells R1, R2 and R4 from 1992 to 2019. The wells have been placed along the groundwater flow direction in an increasing distance from the edge of the landfill.

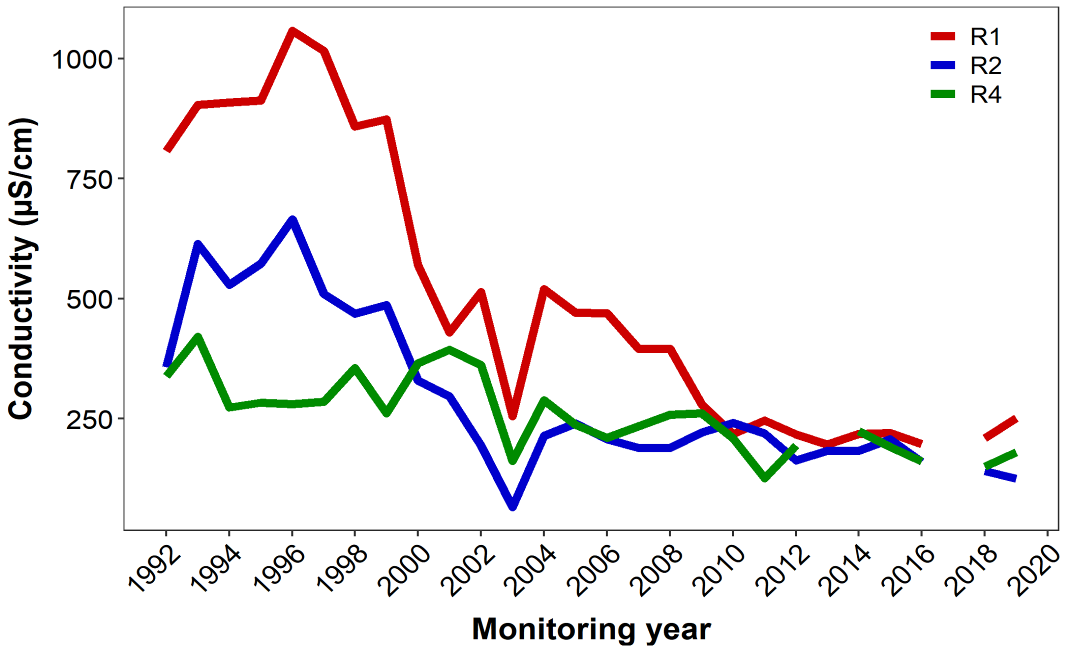

Figure S3. Long-term changes in annual mean values of conductivity across the sampling wells R1, R2 and R4 from 1992 to 2019. The wells have been placed along the groundwater flow direction in an increasing distance from the edge of the landfill.

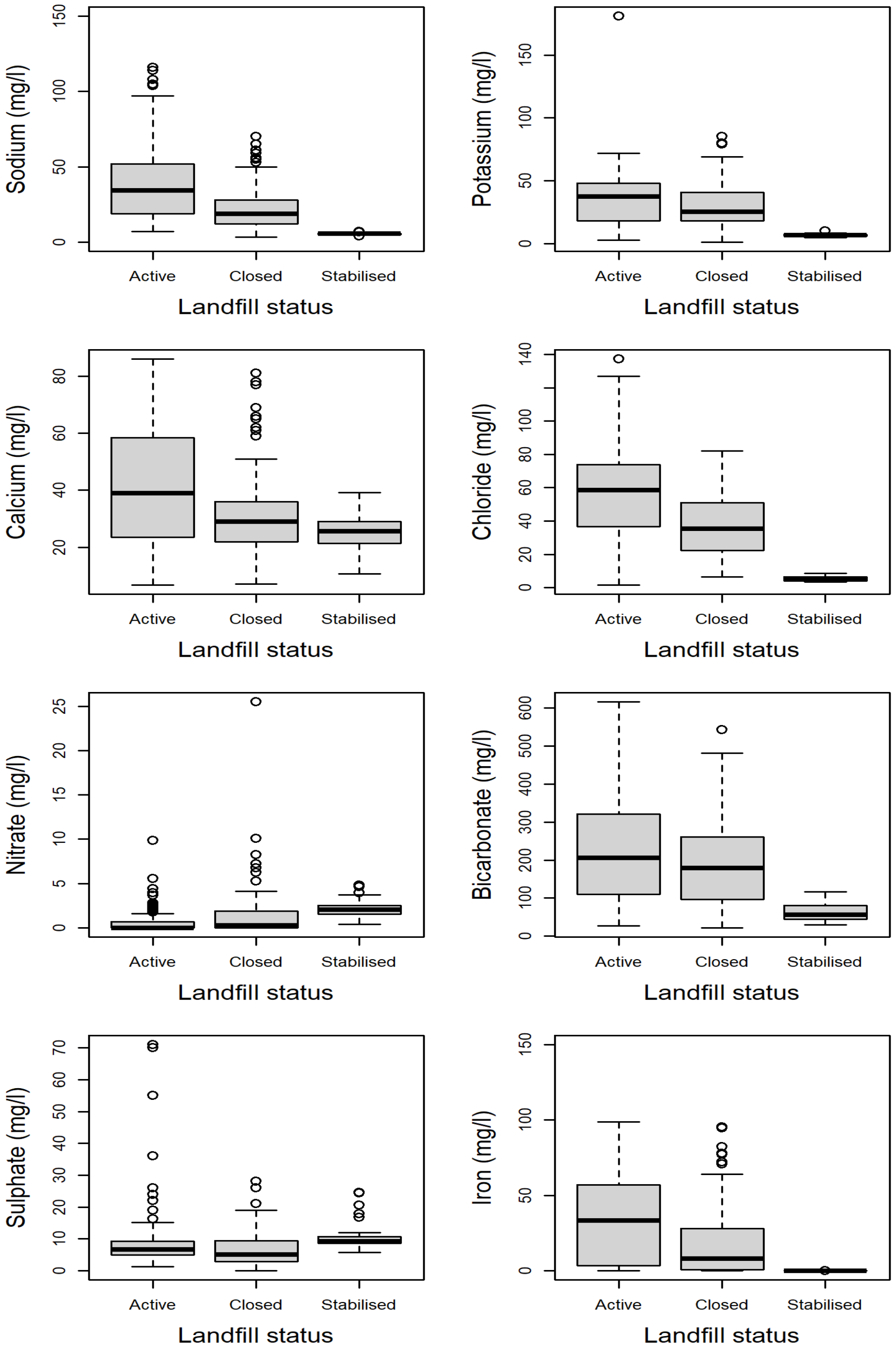

Figure S4. Changes in the groundwater quality as a function of the stages of the landfill. All the parameters indicate significant differences across the different stage. Sodium χ^2 = 167.86, df = 2, p < 2.2e-16; potassium χ^2 = 95.9, df = 2, p < 2.2e-16; calcium χ^2 = 41.3, df = 2, p = 1.093e-09; chloride χ^2 = 164, df = 2, p < 2.2e-16; nitrate χ^2 = 65.6, df = 2, p = 5.541e-15; bicarbonate χ^2 = 80.5, df = 2, p < 2.2e-16; sulphate χ^2 = 45.3, df = 2, p = 1.446e-10; iron χ^2 = 146, df = 2, p < 2.2e-16.

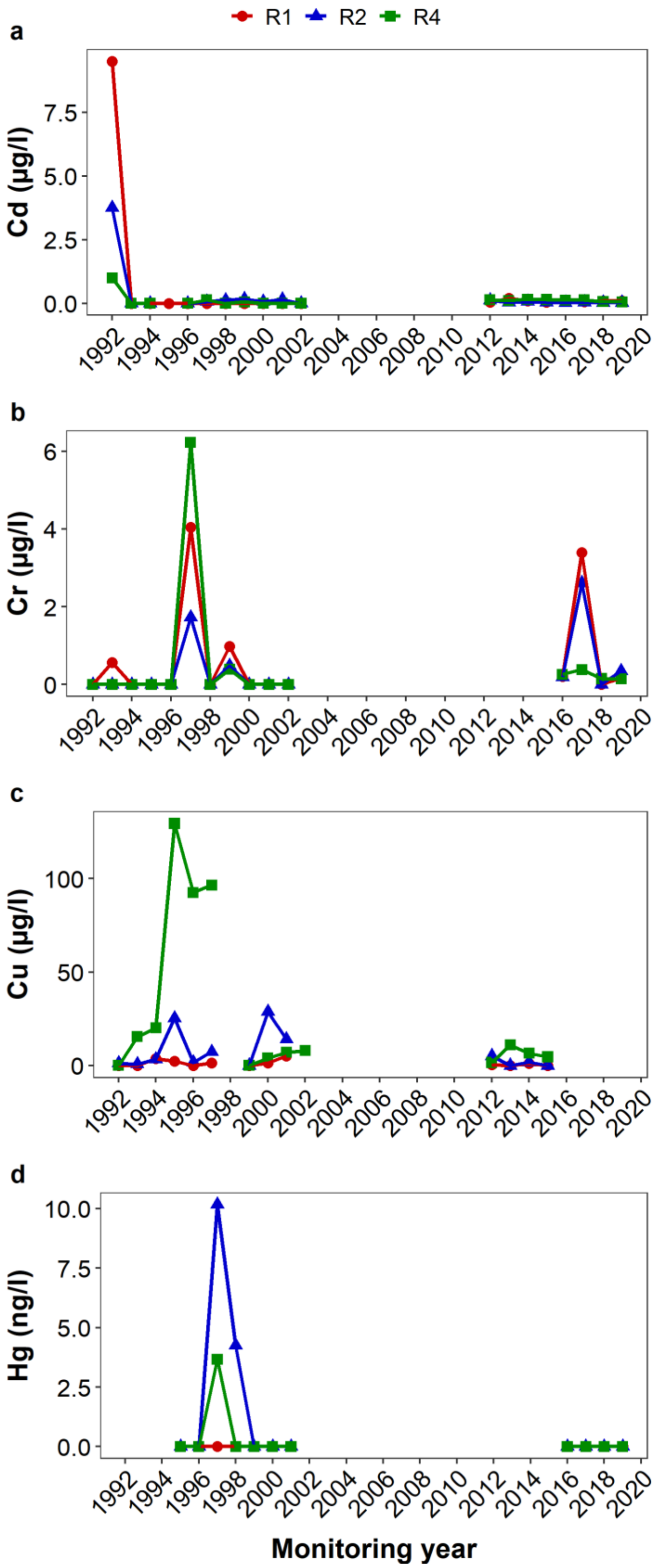

Figure S5. Long-term changes in annual mean values of cadmium (**a**), chromium (**b**), copper (**c**) and mercury (**d**) across the sampling wells R1, R2 and R4 from 1992 to 2019. The wells have been placed along the groundwater flow direction in an increasing distance from the edge of the landfill. all measurements below the limit of detection were treated as zero.

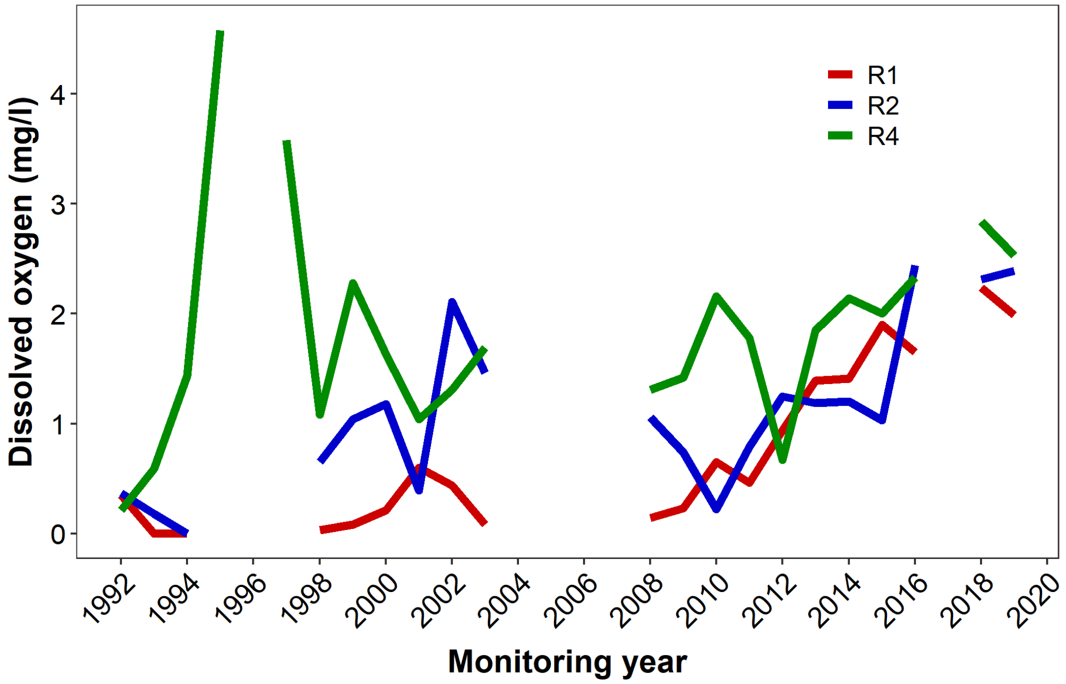

Figure S6. Long-term changes in annual mean values of dissolved oxygen across the sampling wells R1, R2 and R4 from 1992 to 2019. The wells have been placed along the groundwater flow direction in an increasing distance from the edge of the landfill.
